# Photodecoration of tungsten oxide nanoparticles onto eggshell as an ultra-fast adsorbent for removal of MB dye pollutant

**DOI:** 10.1038/s41598-024-65573-5

**Published:** 2024-06-24

**Authors:** Reza Dadashi, Morteza Bahram, Khalil Farhadi, Zartosht Asadzadeh, Javad Hafezirad

**Affiliations:** Department of Analytical Chemistry, Faculty of Chemistry, Urmia University, Urmia, Iran

**Keywords:** Photochemical modification, Eggshell, Dye removal, Methylene blue, WO_3_, Environmental sciences, Chemistry

## Abstract

Nowadays, the use of natural wastes and adsorbents along with their modification by simple and new methods based on metal oxides to remove dye pollutants has been the focus of many researchers. In this study, for the first time, simple and low-cost modification of eggshell (EGS) with tungsten oxide (WO_3_) based on the photochemical modification method as a green, ultra-fast, cost-effective, and biodegradable adsorbent is reported to remove of methylene blue (MB) dye pollutant. The EGS modified by WO_3_ was investigated by EDX, EDX mapping, XRD, FE-SEM, and UV–Vis Diffuse Reflectance (DRS) analyses. The obtained results show that the modified EGS by WO_3_ has more than ten times (78.5%) the ability to remove MB dye pollutant within 3 min compared to bare EGS (11%). Various parameters including dye pollutant pH, dye concentration, adsorbent dosage, and reusability of the WO_3_/EGS adsorbent for removal of MB dye pollutant were investigated and the result show that the adsorbent capacity of WO_3_/EGS is 1.64 mg g^−1^. EGS adsorbent The synthesis of WO_3_/EGS adsorbent with a novel photochemical method as a fast and very cheap adsorbent with excellent efficiency can be a promising alternative adsorbent for various purposes in removing dye pollutants from water environments.

## Introduction

One of the most pressing global challenges is the escalation in water consumption, driven by population growth, urbanization, and industrialization. Daily, these water sources are polluted by industrial effluents, which impairs the discipline of ecosystems and jeopardizes the health of all living organisms^1^. Dyes are a significant category of environmental pollutants that are abundantly present in wastewater generated by various industrial processes, including textile, dyeing, tanning, pulp and paper, paint, and pigments^2^. Methylene blue (MB) is one of the most used dyes in the mentioned industries. While this dye is used in low amounts in the pharmaceutical industry, but its entry into the environment and increases it in water, endangers the health of animals and humans^3^. Also, its presence in aquatic ecosystems causes changes and harmful effects on plants and aquatic in the form of genetic mutations. Therefore, removing MB while improving environmental conditions also brings effective economic effects^4,5^. Different physical^6^, chemical^7^, and biological^8^ methods for removing dyes in water environments including filtration, chemical precipitation, microbial decomposition, reverse osmosis, surface adsorption, and other conventional methods have been reported in the literature^9–11^.

Adsorption is a well-known equilibrium separation process and an effective and applicative method for the removal of different pollutants. This phenomenon has attracted much attention due to its selectivity and high efficiency, cost-effectiveness, availability, and simplicity. By using this method, the pollution present in the water is transferred to the surface of the adsorbent. As a result, the amount of contamination in the wastewater is decreased and the adsorbent that has been contaminated can be retrieved or gathered^12^. The adsorbents are classified into two categories: natural and synthetic. Natural adsorbents are such as activated carbon, clay, by-products, and agricultural waste^13,14^, and macromolecules and resins are classified as synthetic adsorbents^15^. Natural adsorbents are of special importance in this process due to their biodegradability, cost-effective, and availability.

Adsorption efficiency as a surface phenomenon is strongly dependent on the surface chemistry of the adsorbent. Modification of the adsorbent surface with various modifiers, including metal oxides, causes changes in its physical, chemical, and biological properties^16^. The advantages of using metal oxides are their availability, cheapness, and less hazard to the environment. Several reports on the removal of pollutants, such as the removal of arsenic^17^, phenolic compounds^18^, phosphorus and phosphate^19^ as well as wastewater treatment^20^ and other cases, using modified adsorbents by metal oxides are reported in the literature.

So far, many natural adsorbents have been used to remove dye pollutants from wastewater, including tree bark^21^, various plants^22^ and fruit peel^23^, eggshells^24^, etc. Every year, about 8 million tons of eggshells (EGS) waste are produced worldwide, which causes pollution of the human living environment and is an environmental concern^25^. Most of the EGS are made up of calcium carbonate, which is rich in calcium. So far, many articles have been reported on the use of EGS as a very low-cost and readily available adsorbent to remove MB dye pollutants. Tsai et al. have used EGS to remove basic blue 9 and acid orange 51, from aqueous solutions^26^. Elkady et al. used an immobilized EGS with a polymer mixture of alginate and polyvinyl alcohol as a biocomposite adsorbent for the adsorption of C.I. Remazol Reactive Red 198 from aqueous solution^27^. Rajoriya et al. have used EGS to remove methyl red (MR) dye from aqueous solutions^28^. Chou et al. have used EGS to remove copper ions in wastewater^29^. Mobarak et al. synthesized hydroxyapatite from chicken eggshell and used it for removing congo red dye from aqueous solution^30^. Also, EGS has been used as a support substrate for the synthesis of adsorbents to remove dyes^31^.

In recent years, tungsten trioxide (WO_3_) nanoparticles have been widely used for the removal and adsorption of dye pollutants^32–37^ due to their low environmental hazard properties and excellent adsorption and photocatalytic properties^38^. Additionally, WO_3_ exhibits stable structural in harsh environments that making it a promising material for use as an environmentally friendly adsorbent, particularly for the treatment of The perfect substances for adsorption must display exceptional removal abilities, rapid uptake routes, and possess sturdy mechanical structure. Nonetheless, nanoscale WO_3_ particles offer certain benefits, such as the ability to easily control their dimensions, resulting in a high surface area, acceleration of mass transportation, and overall robustness industrial wastewater^37^. In this modification method, Irradiation of UV light on the adsorbent and the metal oxide nanoparticles used in them can excite the electrons on their surface and create oxide layers on the surface of the modified adsorbent, as a result, it can increase the photocatalytic activity and capacity properties of adsorbent for photodegrading or removal of dye pollutants. In this research, for the first time, the WO_3_/EGS adsorbent was synthesized by photochemical modification as a simple, low-cost, and green method. WO_3_/EGS adsorbent was used as an ultrafast, efficient, cheap, readily available, and biodegradable adsorbent with excellent and rapid performance for the removal of MB dye pollutants, and the effective parameter for dye removal was optimized carefully.

## Experimental

### Materials and devices

All materials used were of analytical grade without further purification. Sodium tungstate (Na_2_WO_4_, 99%), hydrogen peroxide (H_2_O_2_, 30%), nitric acid (HNO_3_, 65%), and methylene blue (C_16_H_18_ClN_3_S, 98%) were all purchased from Merck (Darmstadt, Germany). UV lamp (40W Mercury lamp, Philips Netherland). Local chicken egg shells were prepared and used. Centrifuge (VELOCITY 14R) and Grinder (BOMANN, KSW 6501 CB, Germany) were used for the separation and crushing of adsorbent. Absorption spectra were recorded using an Agilent 8453 UV–visible spectrometer (USA) equipped with a 1 cm quartz cell.

### Instrumentations

FT-IR spectra were recorded using a Thermo-Nicolet Nexus 670 Fourier transform infrared spectrometer (USA). A ZEISS Sigma VP scanning electron microscope (Germany) performed the FE-SEM characterization of the fabricated adsorbent for morphological studies. XRD (PHILIPS_PW1730, Netherland) and EDX mapping (TESCAN MIRA3, Czech) analyses were used to investigate the elements. The size of the band gaps of the adsorbent surface was measured by diffuse reflectance spectroscopy (S_4100 SCINCO, South Korea). Metrohm-750 desktop pH meter was used to adjust the pH of the reaction medium. The BET characterization was determined using BET (N_2_ adsorption/desorption isotherms on an AUTOSORB-1-C apparatus).

### Preparation of eggshells (EGS)

The chicken eggshells were obtained from restaurants and kitchens as waste. Eggshells were washed several times with deionized water and boiled (100 °C) to remove impurities. After completely washing the shells, they were dried in the open air at room temperature for 24 h. After that, the dried eggshells were crushed in a grinder for 15 min. Finally, the crushed EGSs are passed through the mesh to obtain the same size and uniform surface area of the EGS and modified by WO_3_ according to previous literature^39^.

### Preparation of WO_3_/EGS

**Figure 1:**
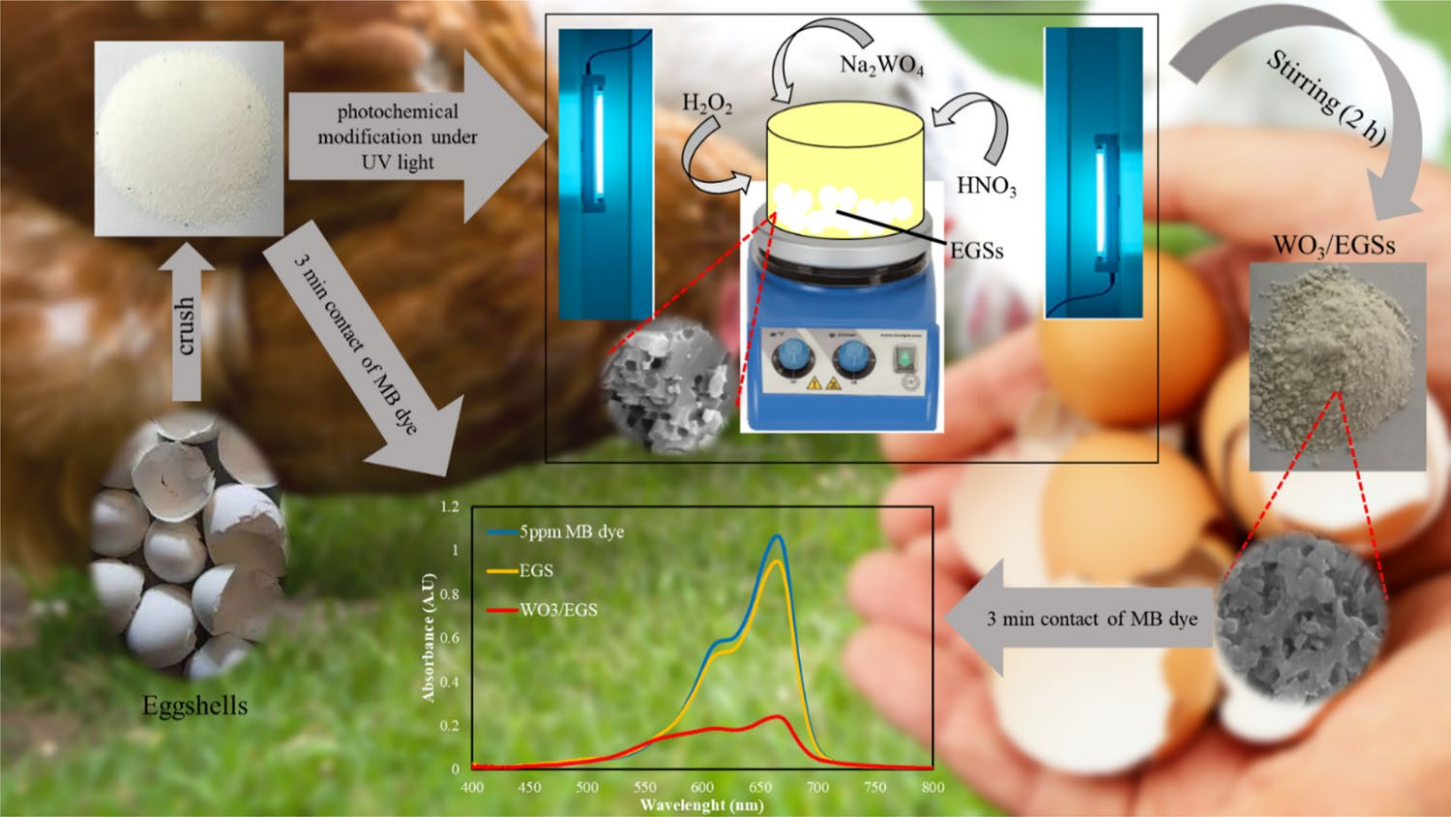
General schematic of the preparation of WO_3_/EGS adsorbent.

To modify the prepared EGS in the previous step with WO_3_, First, the tungsten oxide solution was prepared. for this purpose, in a reaction flask, 2.4 g sodium tungstate was dissolved in 291 mL of deionized water. Then 100 μL of H_2_O_2_ was added to it and the solution was allowed to be stirred for 10 min. In the next step, 9 mL of 3.6 M HNO_3_ was added dropwise to the solution and the reaction mixture was stirred for another 10 min. After the preparation of the sodium tungstate solution, Finally, 3 gr of EGS was added to the reaction flask and placed under the UV light for 2 h to photodeposition of WO_3_ on the adsorbent (The UV distance during the synthesis process was 20 cm). After filtering, the contents of the reaction flask were washed several times with deionized water until the pH value was neutralization and finally dried at 80 °C for 6 h. Figure 1 shows the general schematic of the preparation of WO_3_/EGS adsorbent. The mechanism of the WO_3_ formation process on EGS is reported in reaction (1) and (2):

1 $$2{\text{WO}}_{4}^{2-}+4{\text{H}}_{2}{\text{O}}_{2} \longrightarrow {\text{W}}_{2}{\text{O}}_{11}^{2-}+2{\text{OH}}^{-}+3{\text{H}}_{2}{\text{O}},$$

2 $${\text{W}}_{2}{\text{O}}_{11}^{2-}+10{\text{H}}^{+}+4{e}^{-}\longrightarrow 2{\text{WO}}_{3}+5{\text{H}}_{2}{\text{O}}.$$

### Adsorption experiments

Experiments and examination of methylene blue dye pollutant removal from aqueous solutions were performed at room temperature. MB solution of 100 mg/L was diluted with distilled water to reach a working concentration of 5 mg/L. Experiments were performed at the natural pH of the dye. In the experiment, 20 mL of MB solution was mixed with 0.05 mg of modified adsorbent. After each dye removal, the spectrum of the sample was measured by a spectrophotometer. The removal percentage of MB by WO_3_/EGS at different conditions was calculated using Eq. (3) at a wavelength of 668 nm, that in this equation R is the removal percentage, C_i_ and C_f_ are the initial and final concentrations of MB^39,40^.

3 $$R\%=\frac{{C}_{i}-{C}_{f}}{{C}_{i}}\times 100.$$

Adsorbent capacity (q_t_) calculated from the following equation (Eq. 4)^41^:

4 $${q}_{t}=\frac{{C}_{0}-{C}_{t}}{m}\times V.$$

In this equation (Eq. 4), C_0_ and C_t_ are the initial and final concentrations, V is the volume of the solution (L), m is the weight of the adsorbent, (g) and q_t_ is the adsorption capacity of MB after the contact time of the solution with the adsorbent (mg/g).

### Complying with relevant institutional, national, and international guidelines and legislation

The authors declare that all relevant institutional, national, and international guidelines and legislation were respected.

## Results and discussion

### Characterization

#### Chemical bond characterization of WO_3_/EGS adsorbent

**Figure 2:**
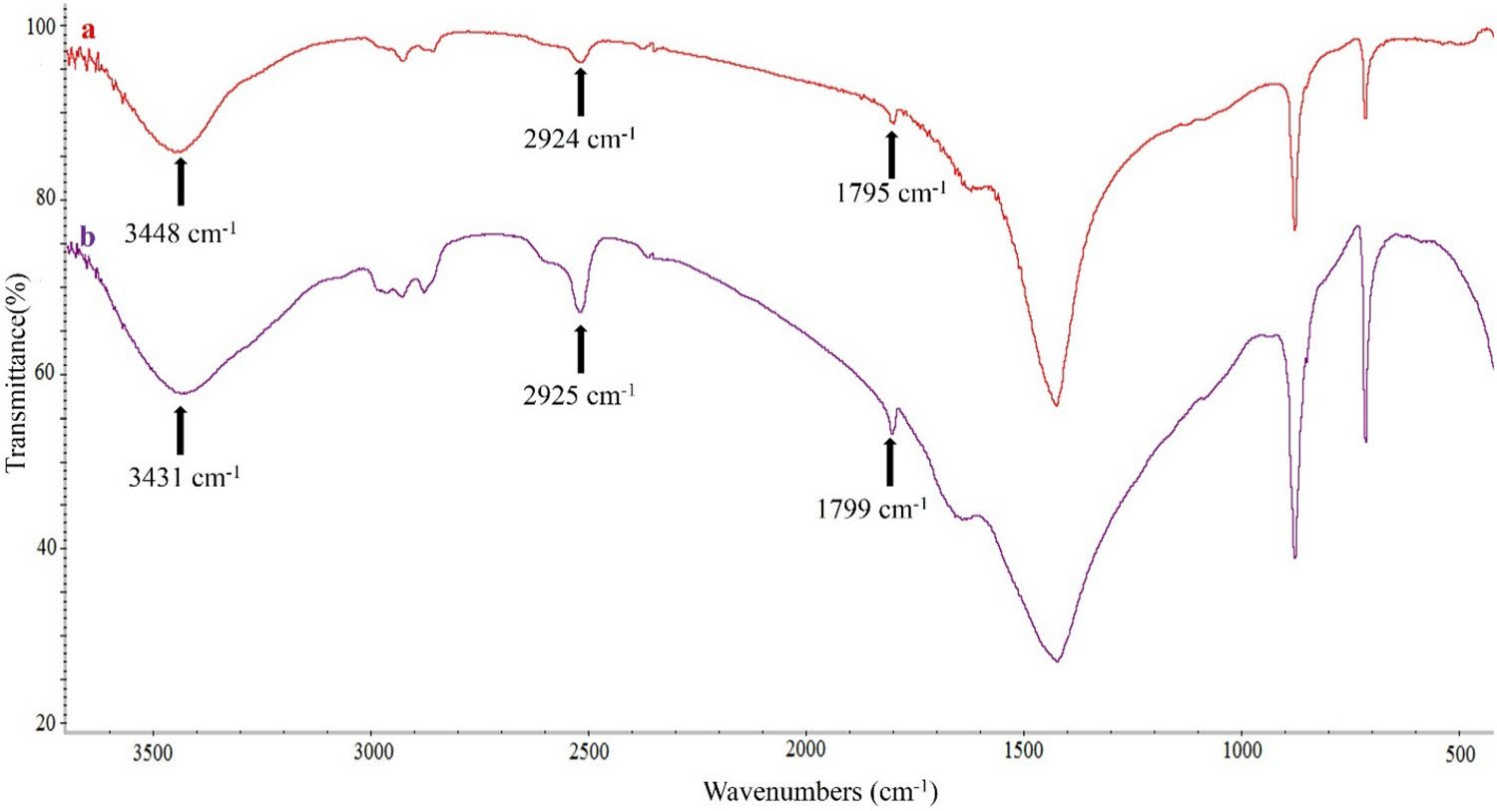
FT-IR spectrum of EGS (a) and WO_3_/EGS adsorbents.

FT-IR analysis was performed to initially prove the modification of the eggshell adsorbent surface with WO_3_ nanoparticles. FT-IR spectra of EGS (a) and WO_3_/EGS (b) adsorbents are shown in Fig. 2. According to the FT-IR spectrum of EGS, the appeared peaks at 1795 cm^−1^, 2515 cm^−1^, and 3448 cm^−1^ are related to carbonyl stretching, hydrogen vibration, and hydroxyl stretching groups, respectively^42^. Figure 2b shows the FT-IR spectra of WO_3_/EGS adsorbent. As shown from this spectra, the peaks that appeared for the EGS have reappeared for WO_3_/EGS with a change in their intensity and with a shift in the wavelength regions, and these changes and the shift of the FT-IR peaks for WO_3_/EGS adsorbent indicate the successful modification of the surface of the EGS by WO_3_. The sharp peak at 873 cm^−1^ in both FT-IR spectra of EGS and WO_3_/EGS has appeared, but the intensity of this peak is higher for the WO_3_/EGS adsorbent, which is due to the stretching vibration mode of the bridging oxygen atoms in the W–O–W functional group for the WO_3_ compound^43^.

#### Surface morphology studies of WO_3_/EGS adsorbent

**Figure 3:**
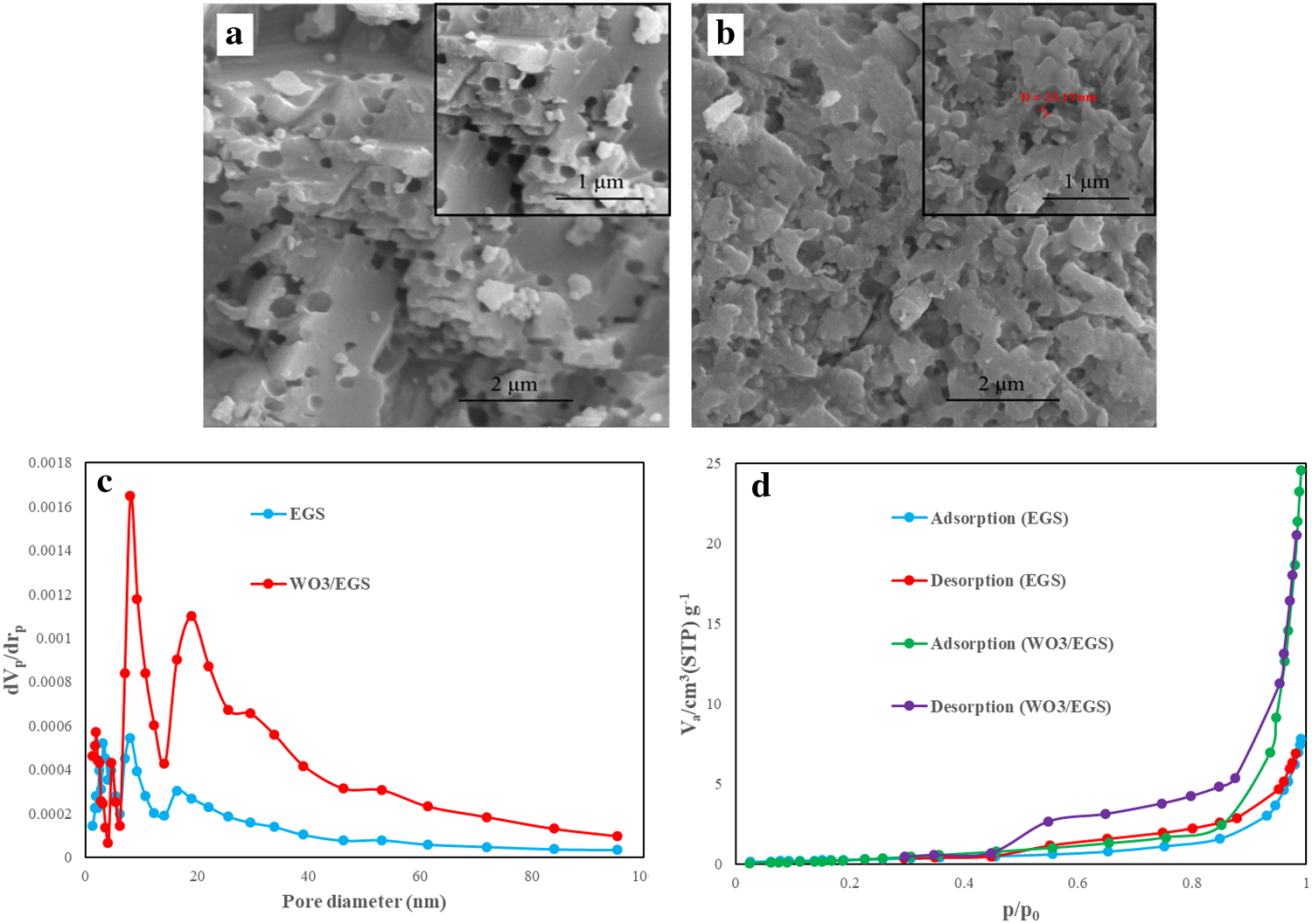
FE-SEM images of (**a**) EGS, (**b**) WO_3_/EGS adsorbents, (**c**) BJH pore size distribution, and (**d**) BET N_2_ adsorption–desorption isotherm of EGS and WO_3_/EGS adsorbents.

Figure 3 shows the surface morphology study of EGS and WO_3_/EGS. The SEM image shows well-defined pore structures. The crystal structure of eggshell particles shows a staggered fracture pattern. Bare EGS has a cheese-like surface with many holes (Fig. 3a). Figure 3b shows the morphology of the WO_3_-modified eggshell, characterized by asymmetric microstructures stacked with primary crystals. The average crystal size with the size distribution shown in the SEM image is about 33.19 nm. The crystallite size distribution histogram shows a greater dispersion of WO_3_ nanoparticles on the surface of EGS. However, surface modification with WO_3_ has been observed to cause changes in surface properties. It is also clear that WO_3_ presents the substrate in a relatively compact form in the pores. Photodecoration of WO_3_ nanoparticles on the EGS surface increases its performance as a adsorbent. by Photodecoration of WO_3_ nanoparticles on the EGS the surface area of it decrease due to the filling of EGS pores by WO_3_ nanoparticles which has already been proven that this decline in surface area is a natural consequence of the accumulation of nanostructures within the pores^44^. Evidence of such a claim was proved by BET analysis. Figure 3c,d show the BET isotherm and BJH (Barrett, Joyner, and Halenda) pore size distribution analysis of the EGS and WO_3_/EGS adsorbent. As shown in these plots and Table 1, the BET surface area was decrease while the BJH pore volume of the EGS after modifying was increased. this enlargement in pore size and volume has confirmed the potential to enhance the ability of WO_3_/EGS in dye removal.

**Table 1:** BET isotherm and BJH pore size distribution analysis of EGS and WO_3_/EGS.

Electrode	BET surface area (m^2^/g)	Mean pore diameter (nm)	BJH pore volume (Cm^3^/g)
EGS	1.0939	43.468	0.012315
WO_3_/EGS	0.71422	209.93	0.038897

#### Structure and crystal characterization of WO_3_/EGS adsorbent

**Figure 4:**
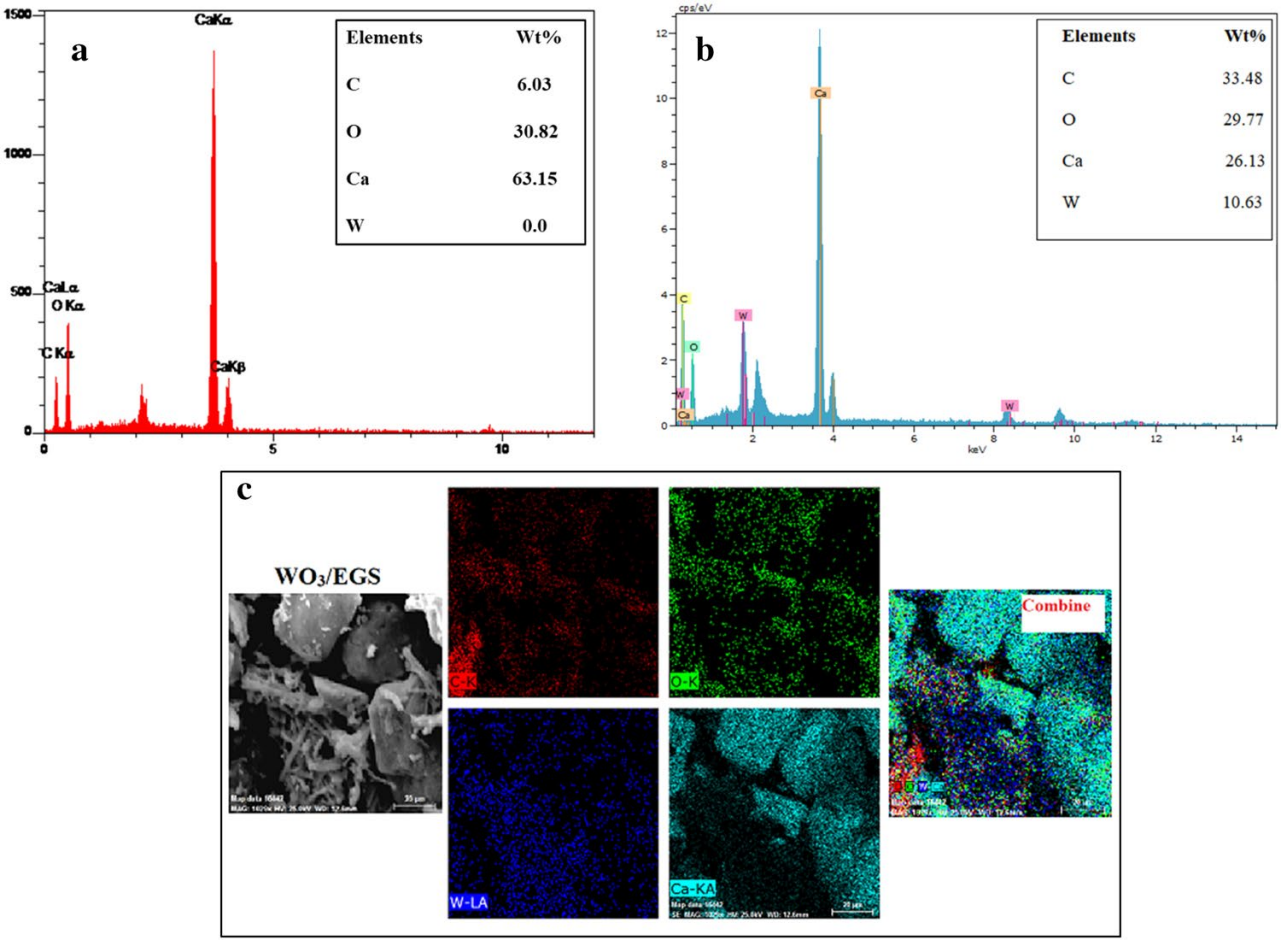
EDX spectrum of EGS (**a**), WO_3_/EGS adsorbent (**b**), and EDX elemental mapping images of WO_3_/EGS adsorbent (**c**).

By energy dispersive X-ray (EDX) analysis, the elements present in the EGS and WO_3_/EGS adsorber were identified (Fig. 4). The results obtained from the related spectra show that there are four elements of carbon (C), calcium (Ca), oxygen (O), and tungsten (W) in the structure of the WO3/EGS sample (Fig. 4b). In contrast, the EGS sample (Fig. 4a) has carbon (C), calcium (Ca), and oxygen (O). The absence of tungsten (W) in the EGS sample compared to the WO_3_/EGS sample indicates that WO_3_ has successfully photodecorated onto EGS in the WO_3_/EGS sample. As can be seen in Fig. 4b, the peak related to the tungsten element was observed at 8.4, 1.8, and 0.2 keV, while these peaks have not appear in the EGS sample spectra. The peak related to oxygen appeared at 0.5 keV. The peak appearing in the range of 0 to 0.3 keV depicts the carbon element and the sharp peak in the 3.6 keV region shows the calcium element as the main structure of the eggshell. The atomic percentage of each of the C, Ca, O, and W elements is shown in a table in the EDX spectrum of each sample. Figure 4c shows the elemental mapping analysis of the C, Ca, O, and W elements for the WO_3_/EGS adsorbent. As can be seen, the distribution of WO_3_ in the EGS is well done which once again confirms the photochemical modification of eggshells by WO_3_.

**Figure 5:**
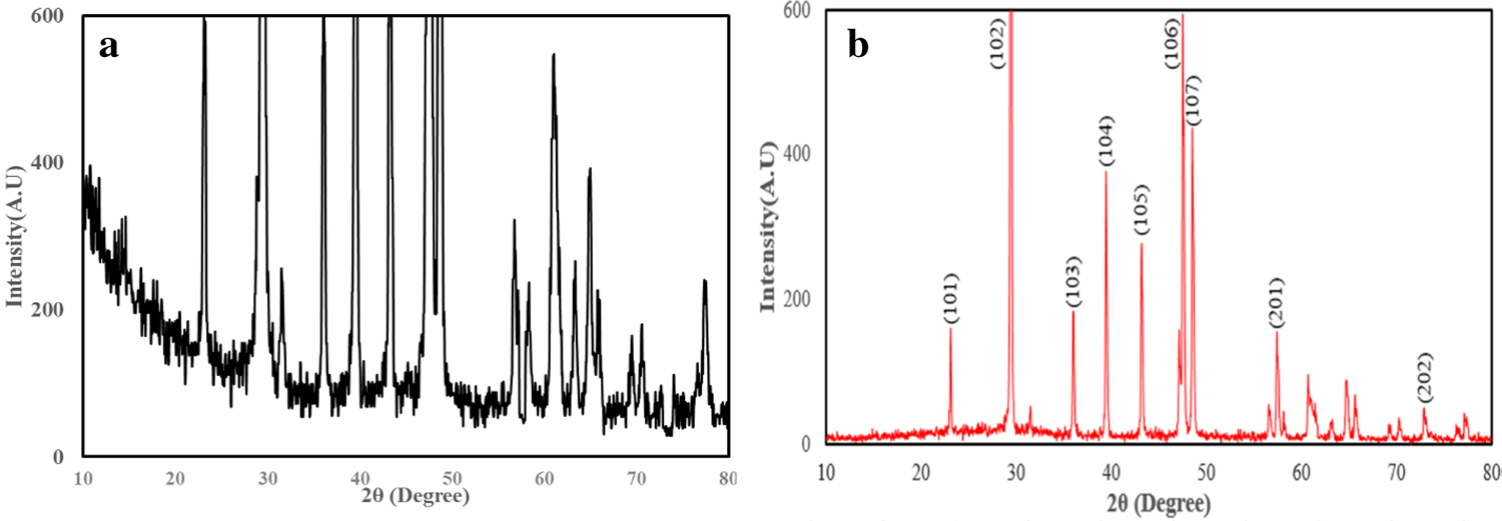
XRD pattern of EGS (**a**) and WO_3_/EGS (**b**) adsorbent.

Figure 5a,b show the XRD pattern of EGS and WO_3_/EGS adsorbent. The XRD analysis results (Fig. 5a) show that the WO_3_/EGS adsorbent like EGS adsorbent has multiple peaks of (101), (102), (103), (104), (105), (106), and (107) which are related to calcium carbonate^45^ and the other typical diffraction peaks ((201) and (202), etc.) are related to the profile of hexagonal WO_3_ nanoparticles^46,47^. Also, the decrease in the intensity of peaks related to EGS after modification with WO_3_ indicates the successful modification of EGS by WO_3_ nanoparticle. In general, the EDX patterns as well as the results of XRD analysis have proved the successful photochemical deposition of tungsten oxide on the eggshell.

#### Investigating the band gap of adsorbers

**Figure 6:**
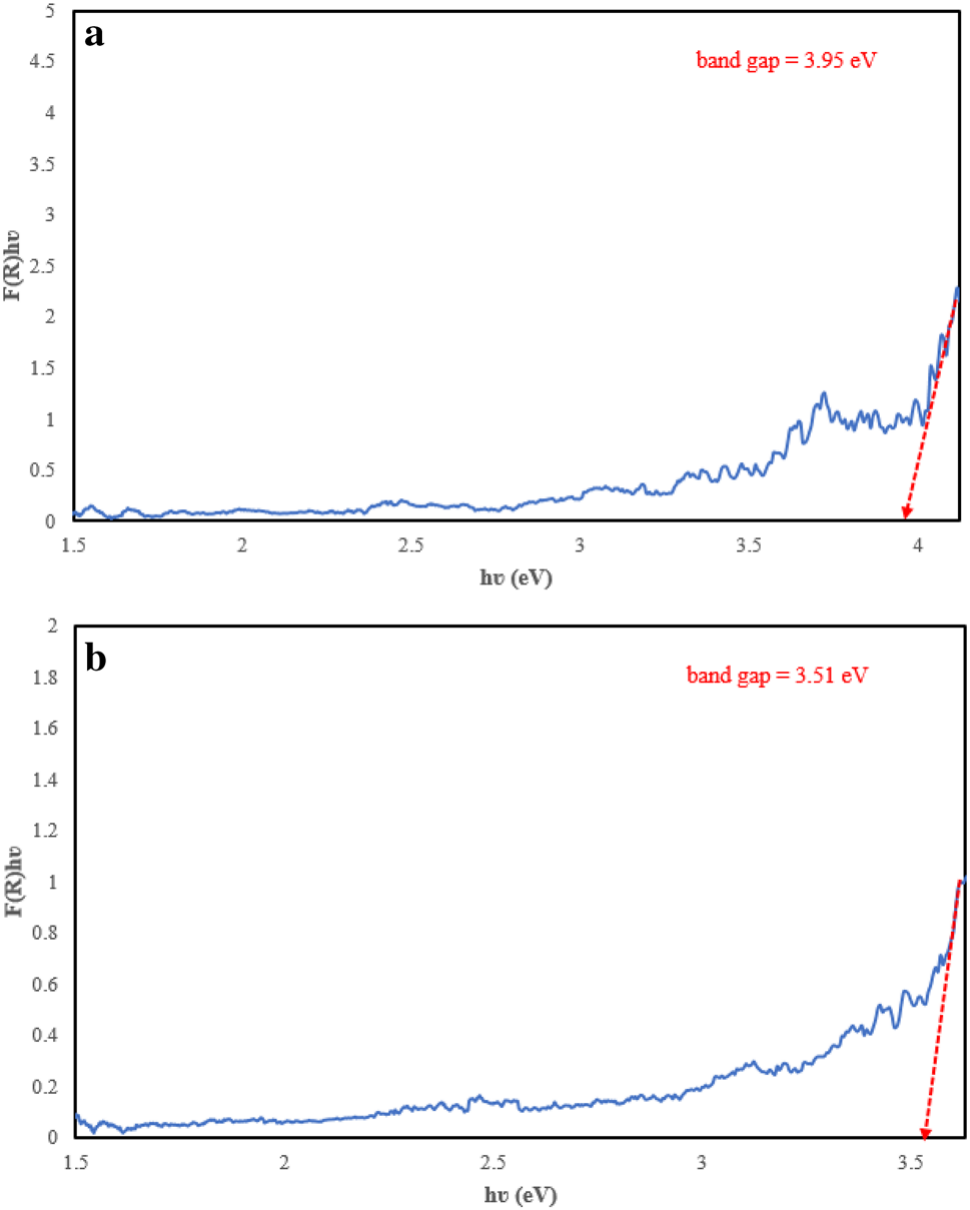
DRS spectrum of EGS (**a**) and WO_3_/EGS (**b**).

The optical properties of EGS and WO_3_/EGS absorbers were investigated by Diffuse Reflectance Spectroscopy (DRS). Figure 6a,b show the band gap obtained from DRS analysis of EGS and WO_3_/EGS. The Kubelka–Munk equation (Eq. 5) was used to convert the reflectance into the equivalent absorption coefficient (α), which is proportional to the Kubelka–Munk function F(R) as follows^48^:

5 $${F\left(R\right)=\left(\frac{{\left(1-R\right)}^{2}}{2R}\times h\nu \right)}^{1/2},$$

where *R* is the measured absolute reflectance of the samples.

The band gap was obtained from plots of [F(R)hν]^1/2^ versus hν, with the intercept of the extrapolated linear part of the plot at [F(R)hν]^1/2^ = 0 representing the band gap.

As shown in Fig. 6, with the modification of EGS by WO_3_ nanoparticles, the band gap of the WO_3_/EGS adsorbent decreases, and this effect can be attributed to the optical property and nanostructure of the WO_3_ compound, which causes such a decrease and leads to an increase in the performance of the adsorbent to remove the MB dye pollutant. Also, the decrease in the value of the band gap (from 3.95 to 3.51 eV) in WO_3_/EGS absorbent once again confirms the photochemical modification of this natural absorbent.

### The investigation of different parameter

In order to improve the removal of MB dye pollutant by WO_3_/EGS adsorbent, the effect of various factors such as pH, contact time, and adsorbent dosage were investigated. Also, the effect of UV light on MB dye removal was investigated, which was not reported in the affecting parameters due to not affecting the dye removal process.

#### Effect of photochemical modification of EGS by WO_3_ on MB removal

**Figure 7:**
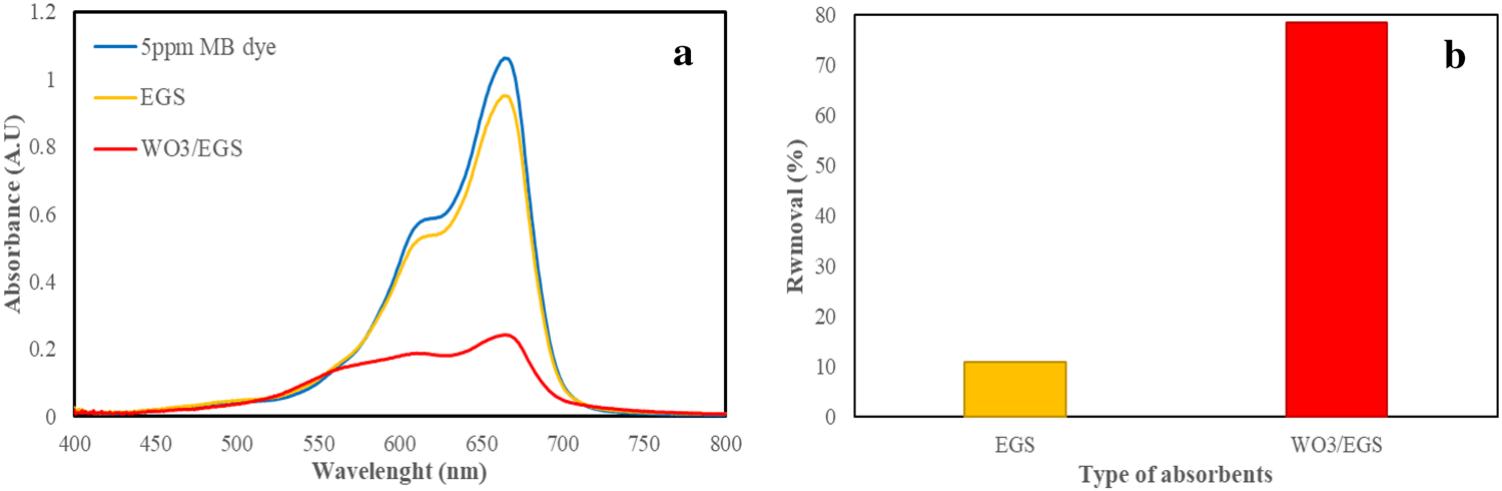
The spectrophotometric spectra (**a**) and removal percentage (**b**) of MB by EGS and WO_3_/EGS ([Adsorbent amount] = 0.05 g, and [MB] = 5 mg/L, 3 min contact of MB dye).

To prove the effect of photochemical modification of WO_3_ on eggshells, first, a constant amount of 0.05 g of EGS and WO_3_/EGS was stirred in 20 mL of 5ppm MB solution for 3 min. After the completion of the reaction time, the spectrophotometric spectra of the MB dye solution were recorded by a UV–Visible spectrophotometer. Figure 7a,b show the spectrophotometric spectra and percentage removal of MB by EGS and WO_3_/EGS. As can be seen in these figures, the spectrum of this comparison shows 78.5% removal and the fast and extraordinary performance of WO_3_ adsorbent in a very short period of 3 min while the EGS adsorber is only able to remove 11% of the MB dye during this time. Accordingly, the Modification of the EGS adsorbent surface with WO_3_ increases its capacity to remove MB dye, which was attributed to the existence of the higher number of surface active sites, available specific surface area, and surface charge in the presence of WO_3_ nanoparticle.

#### Effect of pH and incubation time

**Figure 8:**
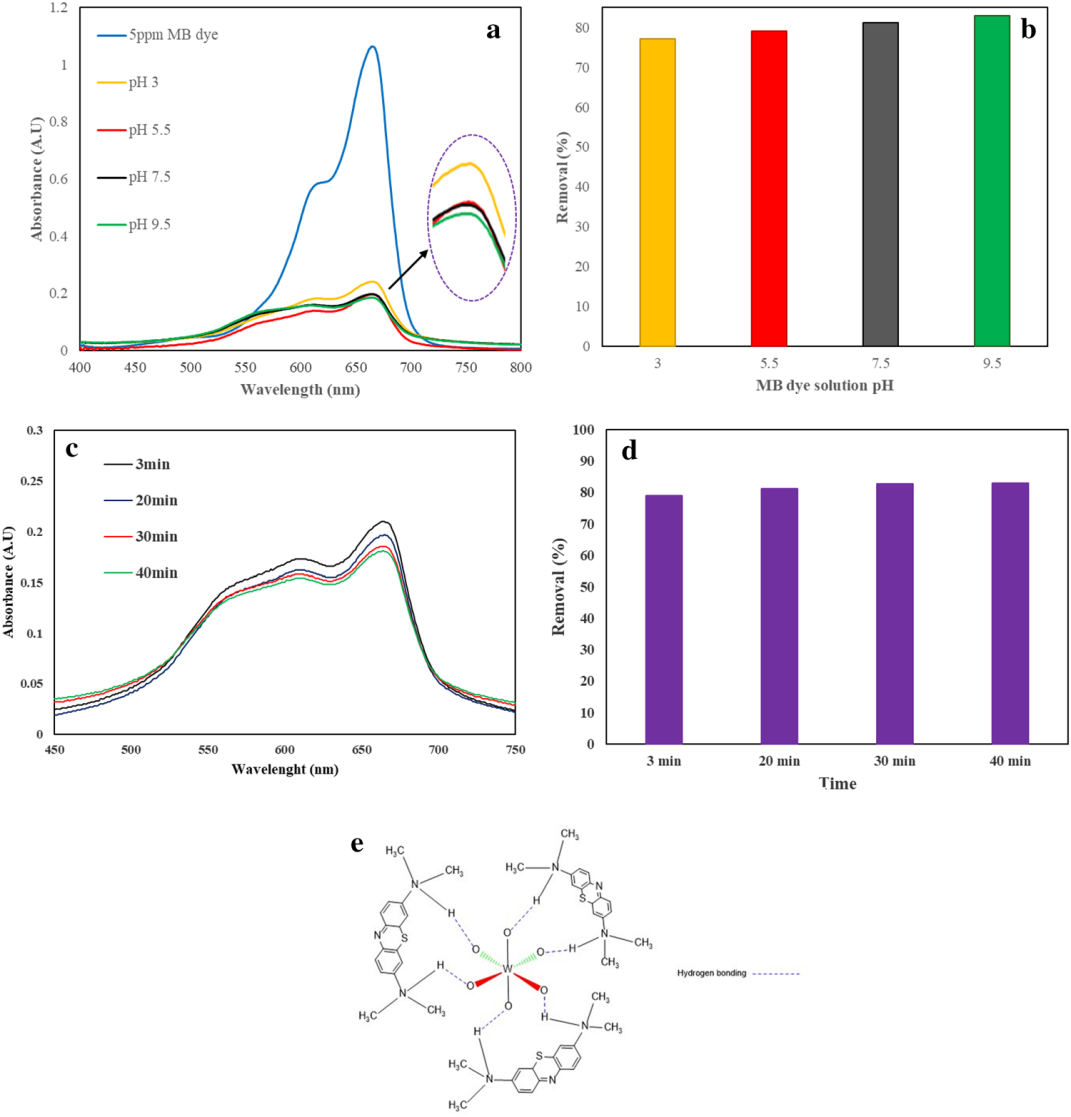
The spectrophotometric spectra (**a**), removal percentage (**b**) of MB dye by WO_3_/EGS in different pH and 3 min incubation of the MB dye pollutant solution, the spectrophotometric spectra (**c**), removal percentage (**d**), and the possible mechanism of MB dye removal (**e**) at different incubation time ([Adsorbent amount] = 0.05 g, and [MB] = 5 mg/L).

Investigating the effect of dye pollutants pH is an important issue in the removal of these pollutants from the wastewater. investigate the effect of the pH of the MB dye pollutant solution on its removal by WO_3_/EGS adsorbent, 20 mL four samples of MB dye solution with a concentration of 5 ppm were prepared at different pH (3, 5.5, 7.5, and 9.5), then 0.05 gr of the WO_3_/EGS adsorbent was added to each of the 4 solutions and were stirred for 3 min. After finished the reaction time, the spectrophotometric spectra and the removal percentage of MB dye by WO_3_/EGS adsorbent were recorded and calculated. Figure 8a,b shows the obtained results of MB dye removal by WO_3_/EGS adsorbent in different pH (3–9.5). As clear from the obtained results, the change of pH does not have a great effect on the removal of this dye pollutant by WO_3_/EGS adsorbent, but as it is also evident in the figure, the percentage of dye degradation is lower in acidic medium. this is probably related to the reason that in acidic medium, strong hydrogen bonds will be formed between excess H^+^ with O–O in WO_3_/EGS, which inhibits the reaction between dye molecules and WO_3_/EGS, and Thus it reduces the removal percentage of dye pollutants. dissolving MB dye in water reduces the pH of the water and brings it to 5.5. Accordingly, in this research, the aim of removal of this dye pollutant from the environment by WO_3_/EGS adsorbent is to remove it at natural pH. Therefore, in this study, for the removal of MB dye pollution by WO_3_/EGS, all affective parameters for dye removal, were optimized in the natural pH (5.5) of the MB dye solution. Finally, after determining the optimal pH, the effect of incubation time was investigated. For this purpose, in optimal conditions and at different incubation times (3, 20, 30, and 40 min), the removal percentage of MB dye pollutant by WO_3_/EGS adsorbent was checked. Figure 8c,d show the spectrophotometric spectra and MB dye removal percentage percentage at different incubation times, respectively. According to the obtained results, there is no significant difference in the MB dye removal percentage by WO_3_/EGS adsorbent after passing more time, which is probably due to the high speed of dye absorption by the active sites on the WO_3_/EGS absorbent. According to the obtained results, the removal percentage for the incubation time of 3 min is not significantly different from the incubation time of 40 min, so the time of 3 min was chosen as the optimal time. Also, the possible mechanism of removal of this dye pollutant by the WO_3_/EGS adsorbent is shown in Fig. 8e.

#### Investigating the effect of adsorbent dosage on MB removal

**Figure 9:**
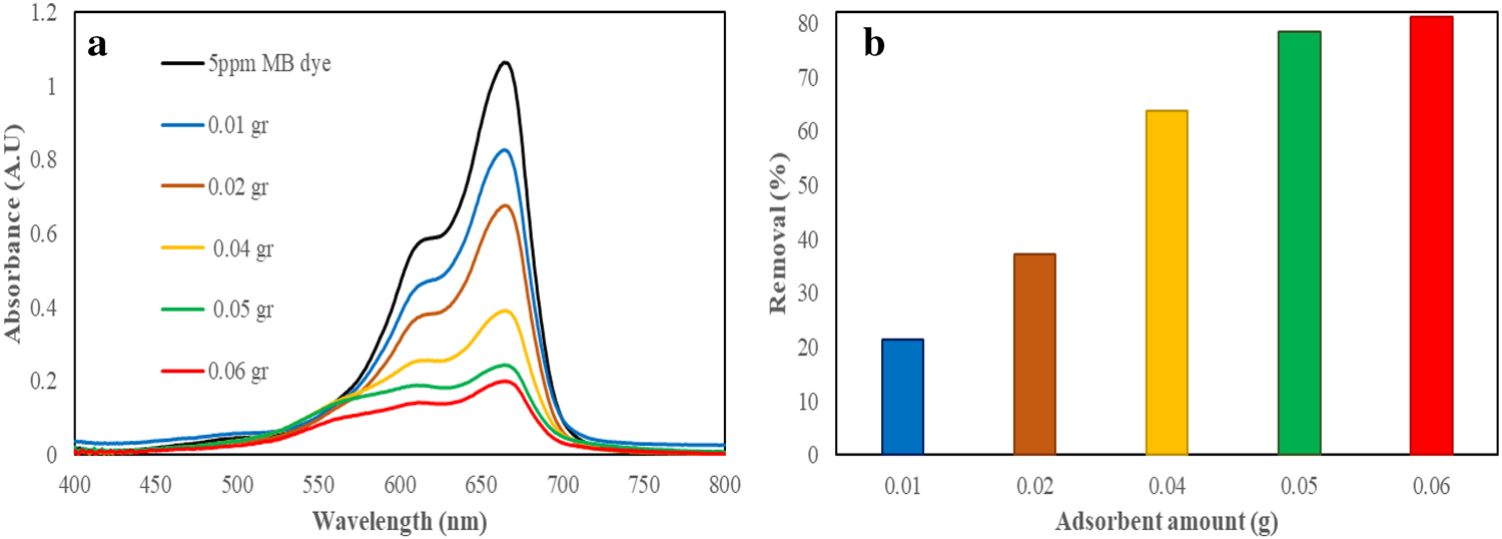
The spectrophotometric spectra (**a**) and removal percentage (**b**) of MB dye by different amounts of WO_3_/EGS adsorbent ([Adsorbent amount] = 0.01–0.06 g, and [MB] = 5 mg/L, 3 min contact of MB dye).

Different weight values of adsorbent were used to select the optimal value of WO_3_/EGS for removal of MB dye pollution. For this purpose, Different amounts of (0.01, 0.02, 0.03, 0.04, 0.05, and 0.06 g) WO_3_/EGS adsorbent were added to the 20 mL MB dye solution with a concentration of 5 ppm and stirred for 3 min. after finished the removal time, the spectrophotometric spectra were recorded and the removal percentage was calculated from Eq. (3). Figure 9a shows the spectrophotometric spectra of MB dye after the removal of it by different dosages of WO_3_/EGS adsorbent. Figure 9b shows a bar chart that the range of MB removal in the presence of 0.01g was calculated from 21.4 to 81.23% for 0.06 g of adsorbent. As clear from this plot, by increasing the adsorbent dose, the removal percentage increases. This increase in removal percentage goes until all the active sites of the WO_3_/EGS adsorbent are available and when the amount of adsorbent exceeds a certain limit due to the lack of complete dispersion of the adsorbent in the environment, the number of available active sites also decreases. Based on this and considering that the percentage of removal in the values of 0.06 and 0.05 g are not much different from each other, so the value of 0.05 g of WO_3_/EGS adsorbent was chosen as the optimal value.

#### Investigating the effect of MB dye concentration for the removal it by WO_3_/EGS

**Figure 10:**
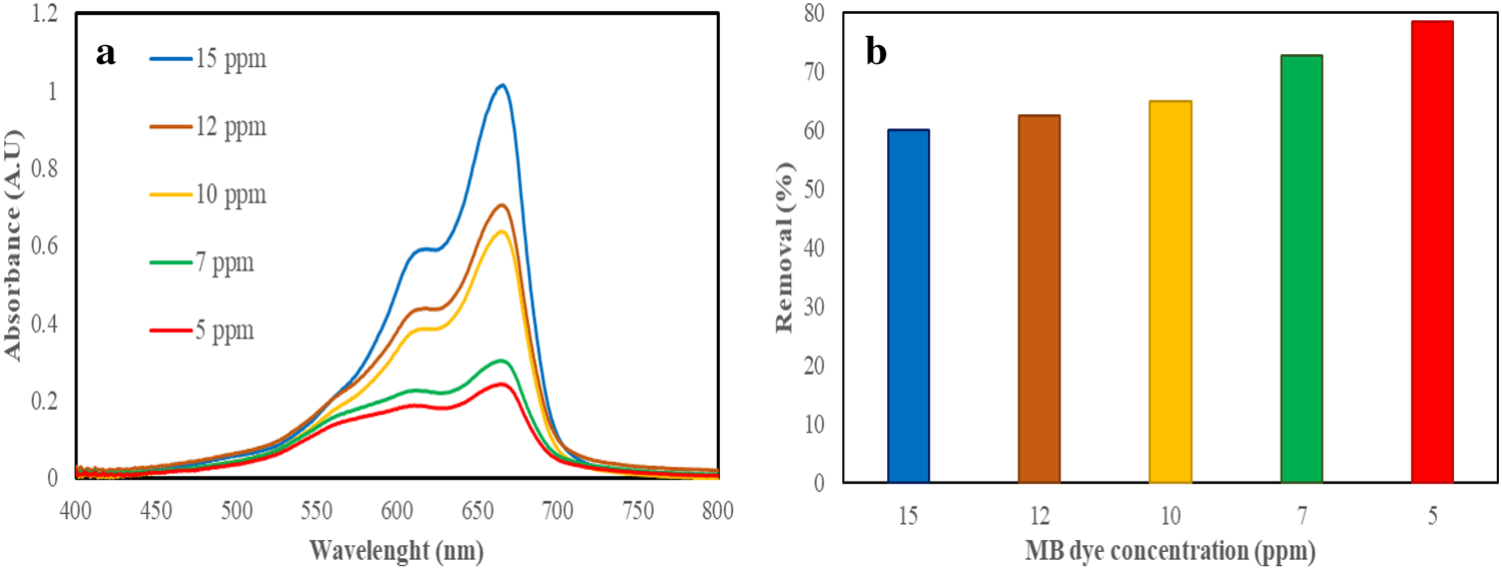
The spectrophotometric spectra (**a**) and removal percentage (**b**) of different concentrations of MB dye by WO_3_/EGS adsorbent ([Adsorbent amount] = 0.05 g, and [MB] = 5–15 mg/L, 3 min contact of MB dye).

One of the main parameters of the research on the removal of dye pollutants is to investigate the effect of the concentration of these pollutants on their removal by adsorbent^49^. Due to the ability of WO_3_/EGS adsorbent to quickly remove MB dye, experiments were performed at higher concentrations. Figure 10a shows the spectrophotometric spectra of different MB dye concentrations (5, 7, 10, 12, and 15 ppm) after 3 min of it removal by 0.05 g WO_3_/EGS adsorbent. Figure 10b show the removal percentage at different concentrations of MB dye pollutant. As it was determined, WO_3_/EGS adsorbent can remove 60, 62.6, 65, 72.7, and 78.5% at concentrations of 15, 12, 10, 7, and 5 ppm, respectively, of MB dye in a short period of 3 min. With the increase in the concentration of dye pollutant, the percentage of its removal by the WO_3_/EGS adsorbent also decreases, which is due to the saturation of all the active sites of the adsorbent. Also, according to the obtained results, it is clear that the WO_3_/EGS adsorbent has a very good adsorption capacity because by increasing the concentration of dye pollutant up to 15 ppm, it still can remove dye pollutant up to 60%. The reason for this decrease in the dye removal percentage with increasing dye concentration can be due to the increase in the number of MB dye molecules in the environment and the constant number of active sites of WO_3_/EGS adsorbent.

#### Reusability of the WO_3_/EGS adsorbent

**Figure 11:**
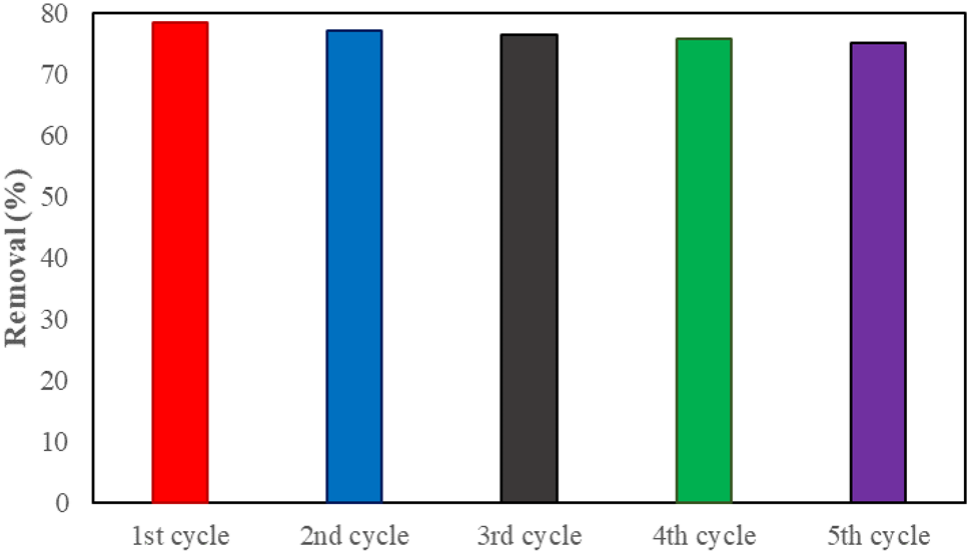
Stability bar graph of MB removal percentage by WO_3_/EGS adsorbent.

The ability to reuse natural and inexpensive adsorbents to remove dye pollutants has been highly regarded. For this purpose, after the first cycle and removing 78.5% of the MB dye, the WO_3_/EGS adsorbent powders were washed several times with an acidic solution of 0.1 mM HCl and then washed with deionized water to neutralize the acidic pH. Finally, it was dried for 24 h at 60 °C in the oven to prepare for the subsequent cycles. As reported in Fig. 11, WO_3_/EGS adsorbent has the ability to remove 77, 76.5, 75.8, and 75% of MB dye during the second to fifth cycles. This shows the extraordinary stability (95.5% after 5 cycle) of WO_3_/EGS adsorbent in short-term 3 min removal of MB dye pollutant. As can be seen, the removal efficiency of MB dye decreased with the increasing number of cycles. The main reason should be that the pollutants adsorbed on the surface of the WO_3_/EGS adsorbent gradually accumulate in each cycle and reduce the active site in each cycle, thereby preventing the interaction between the WO_3_/EGS adsorbent and dye molecules, finally reducing the removal percentage by increasing reuse.

#### Comparison of WO_3_/EGS adsorbent with other natural adsorbents for removal of MB dye pollutant

Table 2 shows the comparison of the removal percentage of MB by other reported natural adsorbents and WO_3_/EGS natural adsorbent. As shown in this table the WO_3_/EGS has an excellent performance as a natural, low-cost, and ecofriendly adsorbent for MB dye removal.

**Table 2:** Comparison performance of WO_3_/EGS for the removal of MB dye with other adsorbents.

Adsorbent	Duration of adsorption (min)	Dye pollutant	Removal (%)	References
Co-Zn BMNPs	15	MB (5 ppm)	43	^50^
Salvia officinalis	5	MB (5 ppm)	60	^51^
MRGO	60	MB (5 ppm)	93	^52^
BMW ZnO NPs	5	MB (5 ppm)	35	^53^
WCFAC	30	MB (5 ppm)	80	^54^
WO_3_/EGS	3	MB (5 ppm)	81.23	This work

## Conclusion

In this study, we modified EGS voids with WO_3_ via photochemical modification as a novel, easy, and cost-effective method. WO_3_/EGS was used as a green adsorbent to remove MB dye. Photochemical modification decreased the band gap of WO_3_/EGS compared to EGS adsorbent. The obtained results from the investigation of different parameters affecting the removal of MB dye pollutants by WO_3_/EGS adsorbent showed that in the presence of WO_3_, this adsorbent has an excellent activity to removal in the natural pH of MB dye after entering surface waters (pH 5.5) so that it can be removal 81.23% of MB dye pollutant in a short period of 3 min. In addition, the WO_3_/EGS adsorbent showed excellent reusability, so after 5 times of using this adsorbent, it showed the ability to remove 75% of the MB dye pollutant. Overall, we believe that this WO_3_/EGS adsorbent and modification method can widely attract the attention of many researchers and be used to remove or destroy chemical and dye pollutants in industrial wastewater in the future.

## Data Availability

The datasets used and/or analyzed during the current study are available from the corresponding author on reasonable request.
